# Effect of basal forebrain somatostatin and parvalbumin neurons in propofol and isoflurane anesthesia

**DOI:** 10.1111/cns.13635

**Published:** 2021-03-25

**Authors:** Shuang Cai, Ai‐Chen Tang, Tian‐Yuan Luo, Shao‐Cheng Yang, Huanhuan Yang, Cheng‐Xi Liu, Yue Shu, Yun‐Chao Pan, Yu Zhang, Liang Zhou, Tian Yu, Shou‐Yang Yu

**Affiliations:** ^1^ Key Laboratory of Brain Science Zunyi Medical University Zunyi China; ^2^ Department of Anesthesiology Affiliated Hospital of Zunyi Medical University Zunyi China; ^3^ Guizhou Key Laboratory of Anesthesia and Organ Protection, Zunyi Medical University Zunyi China

**Keywords:** basal forebrain, isoflurane, parvalbumin‐expressing neurons, propofol, somatostatin‐expressing neurons

## Abstract

**Aims:**

The basal forebrain (BF) plays an essential role in wakefulness and cognition. Two subtypes of BF gamma‐aminobutyric acid (GABA) neurons, including somatostatin‐expressing (GABA^SOM^) and parvalbumin‐positive (GABA^Parv^) neurons, function differently in mediating the natural sleep–wake cycle. Since the loss of consciousness induced by general anesthesia and the natural sleep–wake cycle probably share similar mechanisms, it is important to clarify the accurate roles of these neurons in general anesthesia procedure.

**Methods:**

Based on two transgenic mouse lines expressing SOM‐IRES‐Cre and PV‐IRES‐Cre, we used a combination of genetic activation, inactivation, and chronic ablation approaches to further explore the behavioral and electroencephalography (EEG) roles of BF^SOM^ and BF^Parv^ neurons in general anesthesia. After a single intravenous injection of propofol and the induction and recovery times of isoflurane anesthesia, the anesthesia time was compared. The changes in cortical EEG under different conditions were also compared.

**Results:**

Activation of BF GABA^SOM^ neurons facilitates both the propofol and isoflurane anesthesia, manifesting as a longer anesthesia duration time with propofol anesthesia and a fast induction time and longer recovery time with isoflurane anesthesia. Moreover, BF GABA^SOM^‐activated mice displayed a greater suppression of cortical electrical activity during anesthesia, showing an increase in δ power bands or a simultaneous decrease in high‐frequency power bands. However, only a limited and nuanced effect on propofol and isoflurane anesthesia was observed with the manipulated BF GABA^Parv^ neurons.

**Conclusions:**

Our results suggested that BF GABA^SOM^ neurons play a critical role in propofol and isoflurane general anesthesia, while BF GABA^Parv^ neurons appeared to have little effect.

## Graphical abstract

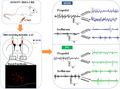

## 1 INTRODUCTION

General anesthetics have been widely used since the introduction of it in the 1840s.^1^ However, few explicit mechanisms have been elucidated that explain how general anesthetics cause a sudden reversible loss of consciousness. The sedation effects of anesthetics, like calmness, drowsiness, and muscle relaxation, are behaviorally similar to the features of endogenous sleep, especially in the non‐rapid eye movement (NREM) period.^2^, ^3^ Moreover, some whole‐brain imaging studies also showed that the state of “unconsciousness” during deep sleep and anesthesia are remarkably similar.^4^ Recently, there has been growing appreciation that neural pathways that regulate the endogenous sleep–wake systems are involved in general anesthesia.^5^, ^6^, ^7^ Thus, several studies on the mechanisms of general anesthesia have focused on the sleep–wake control systems.

The basal forebrain (BF), a large heterogeneous structure in the ventral forebrain, receives projections from the ascending reticular activating system (ARAS) that are then projected to the cerebral cortex.^8^, ^9^ The BF is key to sleep–wake control. BF has three main neuronal populations: cholinergic, glutamatergic, and GABAergic neurons.^10^ Some studies have suggested that stupor or coma can be induced by destroying all these neurons in the BF,^5^ whereas the specific lesion of BF cholinergic neurons produced limited changes in arousal, including in the sleep–wake cycles.^5^, ^11^, ^12^, ^13^ This means the cholinergic neurons are more likely to be related to wakefulness, rapid eye movement (REM) sleep, arousal, and memory than NREM sleep.^11^, ^12^, ^14^, ^15^ Glutamatergic and GABAergic neurons potentially have a critical roles in sleep–wake control.^10^, ^13^ We previously found that the activity of cholinergic neurons in BF could influence propofol and isoflurane anesthesia process.^16^ Furthermore, several studies have indicated that propofol decreases the activity of BF cholinergic neurons via GABA_A_ receptors.^17^, ^18^

GABAergic neurons in the BF are mainly separated into two subtypes with opposite functions: wakefulness‐promoting parvalbumin‐expressing (GABA^Parv^) neurons and sleep‐promoting somatostatin‐expressing (GABA^SOM^) neurons.^10^, ^19^ To clarify their accurate functions in the anesthesia process, we destroyed them respectively and used chemogenetics methods to activate and inactivate the two types of neurons in mice. Moreover, the mice were subjected to behavioral test and electroencephalograph (EEG) under propofol and isoflurane anesthesia. Our findings suggest that GABA^SOM^ neurons in the BF region play a critical role in modulating general anesthesia.

## 2 MATERIALS AND METHODS

### 2.1 Animals

All experimental procedures were approved by the Animal Care and Use Committees of Zunyi Medical University, Guizhou, China, and followed the ARRIVE guidelines and the Guide of Care and Use of Laboratory Animals.^20^ Adult (8–12 weeks, 20–25 g) SOM‐IRES‐Cre (stock N° 018973, Sst^tm2.1(cre)Zjh^/J), and PV‐IRES‐Cre (stock N° 008069, 129P2‐Pvalb^tm1(cre)Arbr^/J) male mice were used in all experiments (provided by Prof. Minmin Luo, National Institute of Biological Science, Beijing, China). Under the control of the SOM/ PV gene promoter, all experimental mice were genotyped by PCR and identified as Cre recombinase. All animals were maintained in an ambient temperature of 23 ± 0.5°C with a relative humidity of 55 ± 2% and 12‐h light/12‐h dark cycle (light on at 8:00 am). Food and water were provided *ad libitum*.

### 2.2 Stereotactic surgery

The mice were anesthetized with pentobarbital (40 mg/kg, intraperitoneal [i.p.]) and then placed on a stereotaxic apparatus (RWD Life Science, Shenzhen, China). Lidocaine (1%) was subcutaneously injected for local anesthesia before exposing the surface of the skull. For lesion experiments, 600 nl (300 nl/side) of virus (rAAV‐CAG‐DIO‐DTA) and an equal volume of saline for the controls were bilaterally injected (speed: 50 nl/min) into BF region (anterior‐posterior [AP]: +0.1 mm, medial‐lateral [ML]: ±1.3 mm, dorsal‐ventral [DV]: −5.4 mm (Paxinos and Franklin, 2001))^21^ of SOM‐IRES‐Cre (*n* = 8) and PV‐IRES‐Cre (*n* = 8) mice, respectively, through a glass micropipette (1‐mm glass stock, tapering to a 10–20 micron tip) using a micro‐syringe pump. The pipette was kept in the region for 10 min to allow the virus to diffuse and was then slowly withdrawn. The electroencephalographic electrodes were placed on the skull (AP: +1.0 mm, ML: ±1.5 mm; AP: −3.5 mm, ML: ±1 mm) simultaneously.^22^, ^23^ The animals underwent further behavioral testing and EEG recording after 3 weeks.

In chemogenetics activation and inactivation experiments, a virus (rAAV‐Ef1α‐DIO‐hM3Dq‐mcherry, hM3Dq /rAAV‐Ef1α‐DIO‐mcherry, control; rAAV‐Ef1α‐DIO‐hM4Di‐mcherry, hM4Di /rAAV‐Ef1α‐DIO‐mcherry, control) was bilaterally and respectively injected into the BF of SOM‐IRES‐Cre and PV‐IRES‐Cre mice (*n* = 8). Next, the EEG electrodes were placed on the skull (AP: +1.0 mm, ML: ±1.5 mm; AP: −3.5 mm, ML: ±1 mm) simultaneously.^22^, ^23^ During all the surgical procedures, a heating pad with a rectal temperature probe was used to keep the body temperature of mice at 37◦C. All experiments were started after 3 weeks.

### 2.3 Experimental procedure

All dosage of behavioral testing and EEG recording were unified. The loss of righting reflex (LORR) and recovery of righting reflex (RORR) time in mice are considered standardized indexes of the induction and emergence time of general anesthesia, respectively. For propofol anesthesia, a single dosage of 20 mg/kg was intravenously injected through the caudal vein with mouse injection fixation devices (Chuangbo Global Biotechnology Co., Ltd., Beijing). The duration of anesthesia, which means the time from LORR to RORR, was recorded. For isoflurane anesthesia, the mice were placed in a recording chamber (RWD Company, Shenzhen, China) filled with 1.4% isoflurane with oxygen at a rate of 1 L/min. The time interval from the start of the isoflurane application to when the mice demonstrated LORR for 30 s was determined as the latency to LORR. The mice were kept anesthetized with 1.4% isoflurane for 30 min and then immediately and gently removed from the chamber. Then, the RORR was quantified in a supine position in room air. The latency to RORR refers to the duration of time before isoflurane stops acting on the mice, and they revert to a prostrate position and landing on all fours.

**FIGURE 1:**
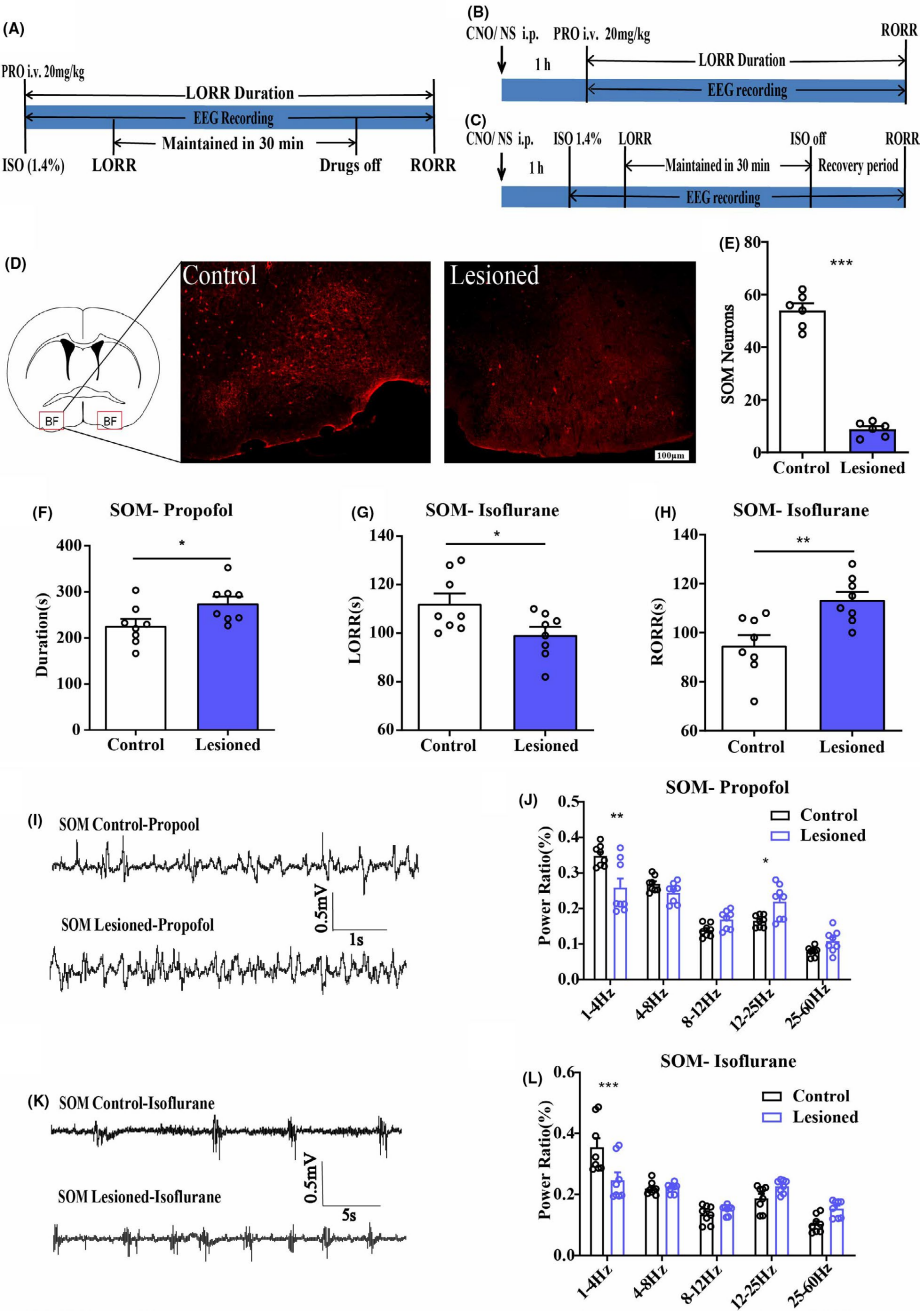
A, The behavioral and EEG recording experiment procedure diagram of neuron lesioned group. B, The behavioral and EEG recording experiment procedure diagram of hM3Dq and hM4Di group under propofol anesthesia. C, The behavioral and EEG recording experiment procedure diagram of hM3Dq and hM4Di group under isoflurane anesthesia. D, The diagram of injection sites (showing by the red boxes) of AAV‐CAG‐DIO‐DTA virus or saline in BF. And immunoflurescence of BF GABASOM neurons in control (left) and lesioned mice (right), scale bar: 100 μm. E, Mean numbers of BF GABASOM neurons in control and lesioned mice (54.00 ± 2.6 to 8.83 ± 2.88). F, The duration time of propofol anesthesia in BF GABASOM neuron lesioned group, *n* = 8, *p* < 0.05, unpaired *t*‐test. G, H, The LORR (G) and RORR (H) time of isoflurane in BF GABASOM neuron lesioned group, *n* = 8, *p < 0.05, **p < 0.01, unpaired *t*‐test. I, Representative EEG traces under propofol anesthesia in BF GABASOM neuron lesioned and control mice. J, The power spectrum analysis of EEG recording in single dose propofol anesthesia of BF GABASOM neuron lesioned group, *n* = 8, *p < 0.05, **p < 0.01, two‐way ANOVA. K, Representative EEG traces under isoflurane anesthesia in BF GABASOM neuron lesioned and control mice. L, The power spectrum analysis of EEG recording under 30 min isoflurane anesthesia in BF GABASOM neuron lesioned group, *n* = 8, **p < 0.01, ***p < 0.001, two‐way ANOVA. All graphs show mean ± SEM

In the lesioned group, we recorded the duration and EEG under propofol anesthesia, as well as LORR, RORR, and EEG under isoflurane anesthesia (Figure 1A). In chemogenetics groups, Clozapine N‐oxide (CNO) (1 mg/ml, 1 mg/kg, i.p.) or saline (0.9%, equal volume, i.p.) were injected randomly 1 h before behavioral testing and EEG recording. The duration and EEG were recorded under propofol anesthesia, as well as LORR, RORR, and EEG were recorded under isoflurane anesthesia (Figure 1B,C). During all tests, a heating pad with a rectal temperature probe was used to keep the mouse body temperature at 37◦C. All mice were sacrificed and subjected to immunofluorescence to verify viral expression and specific transfection after all the experiments were performed. All experiments were performed between 7:00 _AM_ and 6:00 _PM_.

### 2.4 EEG recording and spectral analyses

Electroencephalography signals were captured during all the experimental procedures using a neuronal recording system (Appolo, Bio‐Signal Technologies, USA). These were then digitized and analyzed using the Spike2 software package (Cambridge Electronic Design, Cambridge, United Kingdom). Delta (δ), theta (θ), alpha (α), beta (β), gamma (γ), and total spectral powers were calculated using the frequency bands 1–4, 5–8, 9–12, 13–25, 26–60, and 1–60 Hz, respectively. Relative powers were calculated by dividing the averaged signal power across the frequency range of each band by the total power in 1–60 Hz. Furthermore, GraphPad Software was used for the statistical analysis.

### 2.5 Perfusion and immunofluorescence

All mice were deeply anesthetized with pentobarbital for the perfusion of the phosphate‐buffered saline (PBS), followed by 4% paraformaldehyde (PFA). The brains were removed and post‐fixed in PFA overnight at 4°C and put in 30% sucrose in PBS at 4°C until they sank. The brains were coronally sectioned into 30‐μm slices using a cryostat (Leica CM1950).

The hM3Dq and hM4Di expressing mice were injected with CNO (1 mg/ml, 1 mg/kg, i.p.) or saline (0.9%, equal volume, i.p.), and then kept in their home cage for 2 h before perfusion.

For immunofluorescence, the brain sections were first incubated in blocking solution (PBS containing 2.5% normal goat serum, 1.5% bovine serum albumin, and 0.1% Triton™ X‐100) for 2 h at room temperature. The sections were then incubated with the primary antibody (c‐Fos staining, rabbit anti‐c‐Fos, 1:500, Synaptic Systems; SOM staining, sc‐74556, 1:100, SantaCruz; PV staining, ab104224, 1:1000, Abcam) in a blocking solution overnight at 4°C and washed with PBS. The sections were then incubated with the secondary antibody (goat anti‐rabbit Alexa 594 and Alexa 488, 1:1000, Invitrogen; goat anti‐mouse, Alexa 488, Invitrogen, 1:1000) at room temperature for 2 h. After another wash with PBS, the sections were mounted on glass slides and cover‐slipped with a mounting media (Gold antifade reagent with DAPI, Life Technologies, USA). All images were captured on the virtual microscopy system (Olympus BX63).

### 2.6 Cell counting

Image‐Pro Plus software was used to calculate the number of neurons. The numbers of neurons were quantified by counting positive cells in a 0.6 × 0.6 mm counting box, and 4–6 sections at anatomically matched positions in 100 μm scare bar images (approximately from bregma 0.5 to −0.5 mm, including the horizontal limb of the diagonal band of Broca, magnocellular preoptic nucleus, and substantia innominate, (Paxinos and Franklin, 2001)). The percentage of c‐fos expression in the chemogenetics experimental groups was calculated by the number of c‐fos‐positive cells divided by the number of mcherry positive cells. The results were obtained from six mouse brains from each group.

### 2.7 Statistical analysis

The statistical analysis was performed using commercial software (GraphPad Prism; GraphPad Software). All data were subjected to Kolmogorov–Smirnov tests for normality. Unpaired student's *t*‐tests were to detect all behavioral differences between the lesioned and control groups and cell counts. The differences in the chemogenetics behavioral recording experiments (hM3Di‐Saline and hM3qi‐CNO; hM4Di‐Saline and hM4Di‐CNO) were detected by paired *t*‐test. A two‐way analysis of variance (ANOVA) was used to analyze the EEG recordings. For all results, significant threshold was placed at *p < 0.05, **p < 0.01, ***p < 0.001, and *p* > 0.05 was considered non‐significant (n.s.). All data were shown as mean ± SEM.

## 3 RESULTS

### 3.1 Lesion of the BF GABA^SOM^ and GABA^Parv^ neurons in propofol and isoflurane anesthesia

**FIGURE 2:**
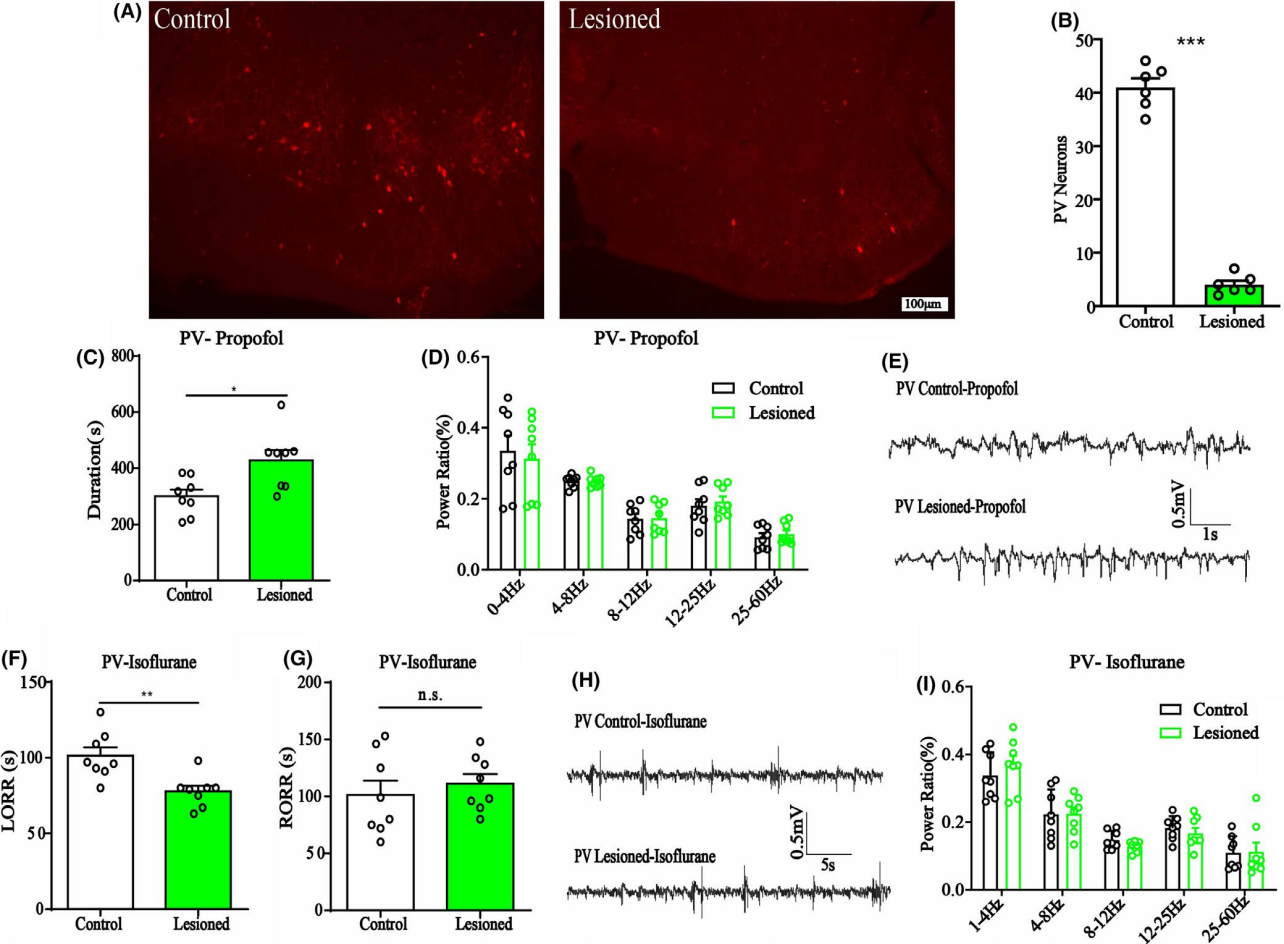
A, Immunoflurescence of BF GABAParv neurons in control (left) and lesioned mice (right), scale bar: 100 μm. B, Mean numbers of BF GABAParv neurons of control and lesioned mice (41.00 ± 1.67 to 4.00 ± 0.73). C, The duration time of propofol anesthesia in BF GABAParv neuron lesioned group, which is longer than that in control mice, *n* = 8, *p < 0.05, unpaired *t*‐test. D, The power spectrum analysis of EEG recording in propofol anesthesia of BF GABAParv neuron lesioned group, *n* = 8, n.s., no significant, two‐way ANOVA. E, Representative EEG traces under propofol anesthesia in BF GABAParv neuron lesioned and control mice. F, G, The LORR (F) and RORR (G) time of isoflurane anesthesia in BF GABAParv neuron lesioned group, *n* = 8, **p < 0.01, n.s., no significant, unpaired *t*‐test. H, Representative EEG traces under isoflurane anesthesia in BF GABAParv neuron lesioned and control mice. I, The power spectrum analysis of EEG recording under 30 min isoflurane anesthesia in GABAParv neurons lesioned group, no difference, *n* = 8, two‐way ANOVA. All graphs show mean ± SEM

To examine whether the two subtypes of GABA neurons have the same or similar functions in natural sleep and anesthesia, we injected the AAV‐CAG‐DIO‐DTA virus vector bilaterally into the BF of SOM‐IRES‐Cre and PV‐IRES‐Cre mice to destroy the GABA^SOM^ and GABA^Parv^ neurons in the BF separately. The immunofluorescence of SOM (Figure 1D, Figure S1A) and PV neurons (Figure 2A, Figure S2A) demonstrated the successful destruction of the neurons in BF (Figure 1E, SOM‐control 54 ± 2.6, SOM‐lesioned 8.8 ± 1.13, *n* = 6, *p* < 0.005; Figure 2B, PV‐control 41 ± 1.67, PV‐lesioned 4 ± 0.73; *n* = 6, *p* < 0.0001, unpaired *t*‐test). All behavioral tests and simultaneous EEG recordings followed the experimental procedure diagram (Figure 1A). In SOM‐IRES‐Cre mice, the duration time was increased, and the EEG of propofol anesthesia also showed significant change (Figure 1F,I,J, control group 226.3 ± 14.92 s, lesion group 274.8 ± 14.67 s, *n* = 8, *p* = 0.035, unpaired *t*‐test; *δ* bands, 0.348 ± 0.010 to 0.258 ± 0.026, *p* = 0.0047, *β* bands 0.165 ± 0.006 to 0.220 ± 0.017, *p* = 0.023, *n* = 8, two‐way ANOVA). Under isoflurane anesthesia, the LORR time was shorter (Figure 1G, control group 112.2 ± 4.25 s, lesion group 99.27 ± 3.32 s, *n* = 8, *p* = 0.012, unpaired *t*‐test) and RORR time was longer (Figure 1H, control group 94.75 ± 4.28 s, lesion group 113.375 ± 3.31 s, *n* = 8, *p* = 0.0039, unpaired *t*‐test) in the lesion group than in the control group, and the delta (δ) power bands on EEG (Figure 1K,L, δ bands, 0.354 ± 0.031 to 0.247 ± 0.025, *p* < 0.001, *n* = 8, two‐way ANOVA).

In PV‐IRES‐Cre mice, the propofol duration time was increased in the GABA^Parv^ neurons lesion group (Figure 2C, 300.3 ± 23.52 s to 428 ± 36.59 s, *n* = 8, *p* = 0.011 unpaired *t*‐test), while EEG remained unchanged (Figure 2D,E). In isoflurane anesthesia, the LORR time was shorter (Figure 2F, 101.3 ± 5.54 s to 77.75 ± 3.702 s, *n* = 8, *p* = 0.0034, unpaired *t*‐test) and RORR time was comparable between the two groups (Figure 2G, 101.3 ± 12.64 s to 111.3 ± 8.46 s, *n* = 8, *p* = 0.31, unpaired *t*‐test). Furthermore, no differences in the EEG results were found (Figure 2H,I). The facilitated anesthesia resulted in decreased EEG results in the lesion of GABA^SOM^ neuron mice. Another, the slight behavioral changes and no other EEG difference in lesion of GABA^Parv^ neurons mice both promoted us to examine the functions of these neurons with reversible activation and inhibition approaches.

### 3.2 Chemogenetic activation of BF GABA^SOM^ and GABA^Parv^ neurons in propofol and isoflurane anesthesia

**FIGURE 3:**
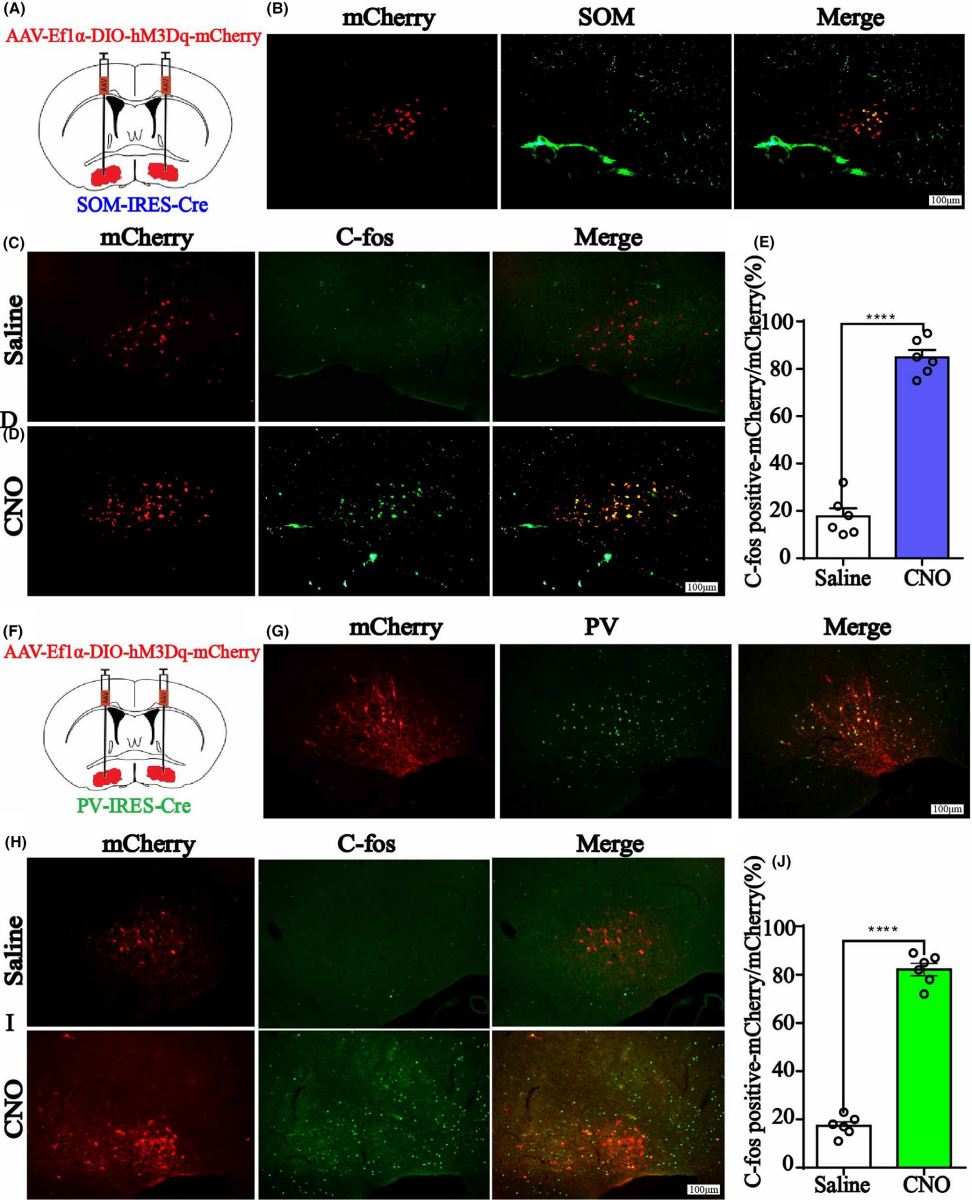
A, The injection sites of AAV‐Ef1α‐DIO‐hM3Dq‐mcherry or AAV‐Ef1α‐DIO‐mCherry vector in BF of SOMIRES‐Cre mice. B, Immunofluorescent of BF GABASOM neurons (green) and hM3Dq virus expression (red), and merged picture. Scale bar: 100 μm. C, D, C‐Fos expression of BF in GABASOM‐hM3Dq mice after saline or CNO i.p. injection. Scale bar: 100 μm. E, The percent of C‐Fos positive cells in BF GABASOM nucleus. Data are presented as mean ± SEM. The percent of C‐Fos expression in GABASOM neurons with CNO pretreatment was significantly higher than that in the saline pretreatment group (*p* < 0.0001, unpaired *t*‐test). F, The injection sites of AAV‐Ef1α‐DIO‐hM3Dq‐mcherry or AAV‐Ef1α‐DIO‐mCherry vector in BF of PV‐IRESCre mice. G, Imnunoflurescent of GABAParv neurons (green) and hM3Dq virus expression (red), and the merged picture. H, I C‐Fos expression of BF in GABAParv‐hM3Dq mice with saline or CNO i.p. injection. Scale bar: 100 μm. J, The percent of C‐Fos positive cells in BF GABAParv nucleus. Data are presented as mean ± SEM. The percent of C‐Fos expression in GABAParv neurons with CNO pretreatment was significantly higher than that in the saline pretreatment group (*p* < 0.0001, unpaired *t*‐test)

**FIGURE 4:**
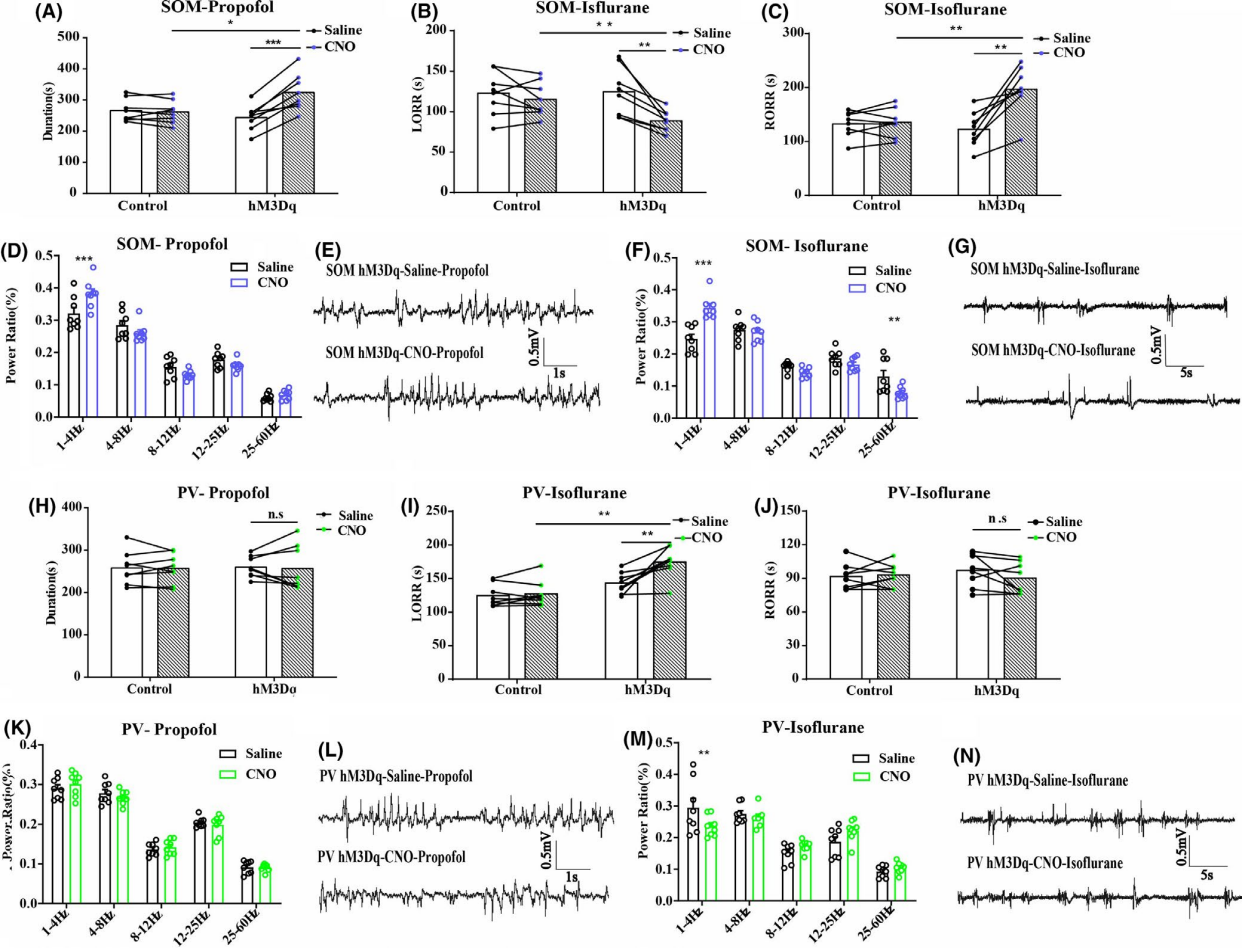
A, The duration time of propofol anesthesia in BF GABASOM neuron with hM3Dq group, *n* = 8, *p < 0.05, ***p < 0.001, paired *t*‐test. B, C, The LORR (B) and RORR (C) time of isoflurane anesthesia in BF GABASOM neuron with hM3Dq group, *n* = 8, **p < 0.01, paired *t*‐test. D, E, The power spectrum analysis (D) and representative EEG traces (E) of EEG recording of mice with activated BF GABASOM neurons in propofol anesthesia, *n* = 8, ***p < 0.001, two‐way ANOVA. F, G The power spectrum analysis (F) and representative EEG traces (G) of EEG recording of mice with activated BF GABASOM neurons in isoflurane anesthesia, *n* = 8, **p < 0.01, ***p < 0.001, two‐way ANOVA. H, The duration time of propofol anesthesia in BF GABAParv neuron with hM3Dq group, *n* = 8, n.s., no significant, paired *t*‐test. I, J, The LORR (I) and RORR (J) time of isoflurane in BF GABAParv neuron with hM3Dq group, *n* = 8, **p < 0.01, n.s., no significant, paired *t*‐test. K–N, The power spectrum analysis of EEG recording of mice with activated BF GABASOM neurons in propofol (K, L) and isoflurane (M, N) anesthesia, *n* = 8, two‐way ANOVA

We injected AAV‐Ef1α‐DIO‐hM3Dq‐mcherry and AAV‐Ef1α‐DIO‐mCherry vector in the BF of SOM‐IRES‐Cre and PV‐IRES‐Cre mice, respectively, to genetically activate the GABA^SOM^ or GABA^Parv^ neurons. Immunofluorescence images validated the virus transfection in the BF of SOM‐IRES‐Cre (Figure 3A,B, Figure S1B) and PV‐IRES‐Cre mice (Figure 3F,G, Figure S2B). C‐Fos expression in BF GABA^SOM^ and GABA^Parv^ neurons with CNO pretreatment was significantly higher than in the saline pretreatment group (Figure 3C–E,H–J,E, the ratio of CNO‐activated BF GABA^SOM^ neurons that transfected on the virus, 17.7%–84.8%, *n* = 6, *p* < 0.0001; J, the ratio of CNO‐activated BF GABA^Parv^ neurons that transfected on the virus, 17.4% to 82.2%, *n* = 6, *p* < 0.0001, unpaired *t*‐test). The duration time of propofol was prolonged in SOM neurons‐activated mice (Figure 4A, control group 266.25 ± 12.66 s to 262.0 ± 13.04 s, *p* = 0.82; hM3Dq group 243.63 ± 14.32 s to 324.25 ± 21.06 s, *p* = 0.0069, *n* = 8, paired *t*‐test). However, no difference in the PV neurons‐activated group under propofol anesthesia was found (Figure 4H, control group 257.88 ± 13.72 s to 257.13 ± 12.31 s, *p* = 0.97; hM3Dq group 260.05 ± 8.86 s to 256.64 ± 18.77 s, *p* = 0.87, *n* = 8, paired *t*‐test). In isoflurane anesthesia, the GABA^SOM^ neurons‐activated mice took less time to be anesthetized (Figure 4B, LORR, control group 122.88 ± 9.52 s to 115.25 ± 7.58 s, *p* = 0.54; hM3Dq group 124.63 ± 10.68 s to 88.50 ± 4.61 s, *p* = 0.0078, *n* = 8, paired *t*‐test) and longer time to recovery (Figure 4C, RORR, control group 132.50 ± 8.38 s to 135.38 ± 9.23 s, *p* = 0.82; hM3Dq group 122.37 ± 11.55 s to 196.88 ± 15.64 s, *p* = 0.0018, *n* = 8, paired *t*‐test). In contrast, GABA^Parv^ neurons‐activated mice were challenging to anesthetize (Figure 4I, LORR, control group 124.50 ± 5.83 s to 127.13 ± 6.81 s, *p* = 0.77; hM3Dq group 143.13 ± 5.65 s to 174.50 ± 7.97 s, *p* = 0.0063, *n* = 8, paired *t*‐test). However, no difference in recovery period under isoflurane anesthesia was observed (Figure 4J, RORR, control group 91.63 ± 4.12 s to 93.00 ± 3.62 s, *p* = 0.81; hM3Dq group 97 ± 5.19 s to 90.13 ± 5.00 s, *p* = 0.36, *n* = 8, paired *t*‐test). The simultaneous cortical EEG also showed significant alterations in the GABA^SOM^‐activated group (Figure 4D–G, SOM‐Propofol, 0.32 ± 0.02 to 0.38 ± 0.04, *p* < 0.001; SOM‐Isoflurane, 0.24 ± 0.04 to 0.31 ± 0.04, *n* = 8, two‐way ANOVA test), but the EEG changes in GABA^Parv^ neuron‐activated group were not obvious (Figure 4K–N, PV‐Propofol, 0.29 ± 0.03 to 0.30 ± 0.04, *p* = 0.96; PV‐Isoflurane, 0.29 ± 0.03 to 0.24 ± 0.01, *p* = 0.0016, *n* = 8, two‐way ANOVA test). These results suggest that activation of the GABA^SOM^ neurons in the BF promotes propofol and isoflurane anesthesia, while activation of the GABA^Parv^ neurons has a little effect.

### 3.3 Chemogenetic inactivation of BF GABA^SOM^ and GABA^Parv^ neurons in propofol and isoflurane anesthesia

**FIGURE 5:**
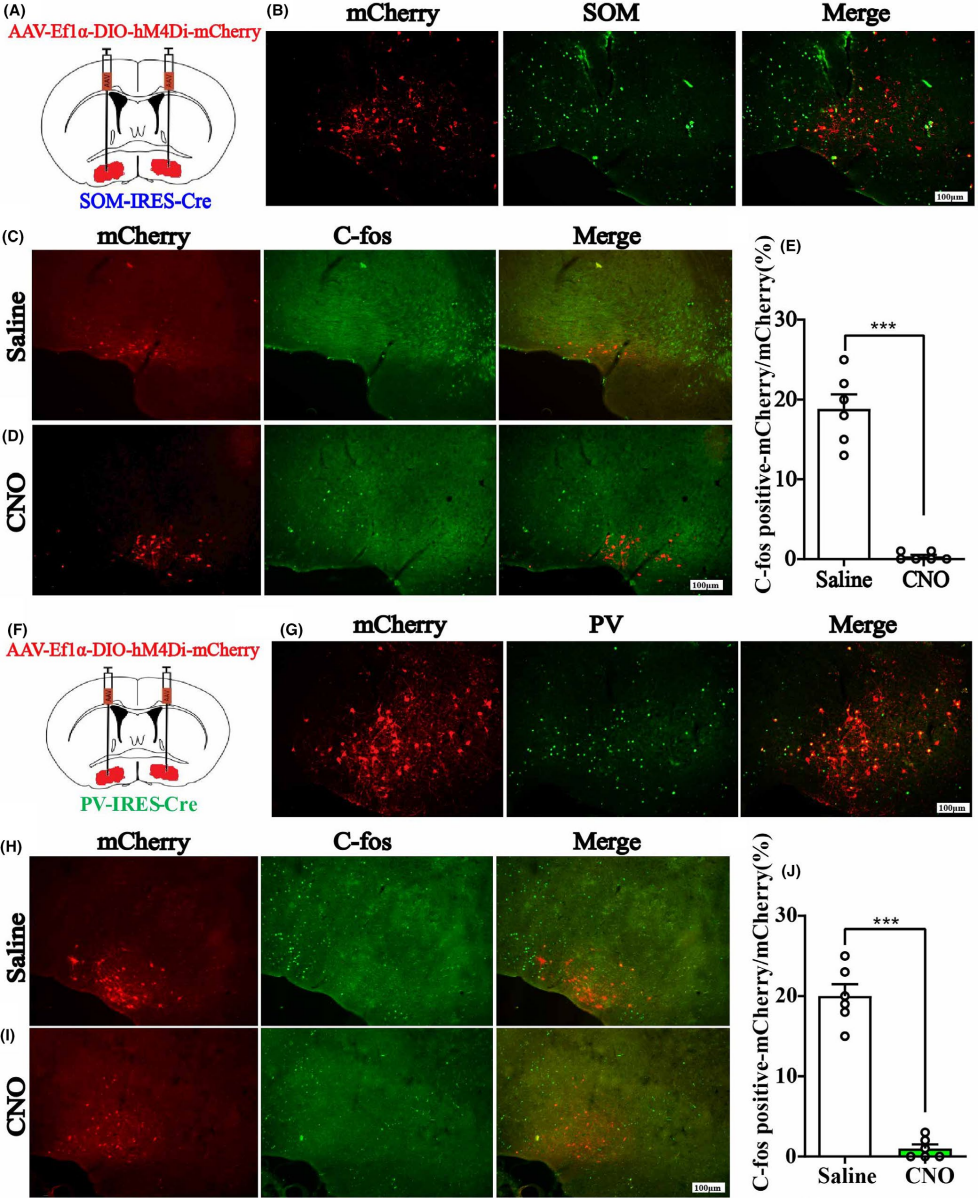
A, The injection sites of AAV‐Ef1α‐DIO‐hM4Di‐mcherry or AAV‐Ef1α‐DIO‐mCherry vector in BF of SOM‐IRESCre mice. B, Immunofluorescent of BF GABASOM neurons (green) and hM4Di virus expression (red), and the merged picture. Scale bar: 100 μm. C, D, C‐Fos expression of BF in GABASOM‐hM4Di mice after saline or CNO i.p. injection. Scale bar: 100 μm. E, The percent of C‐Fos positive cells in BF GABASOM nucleus. Data are presented as mean ± SEM. The percent of C‐Fos expression in GABASOM neurons with CNO pretreatment was significantly lower than that in the saline pretreatment group (*p* < 0.005, unpaired *t*‐test). F, The injection sites of AAV‐Ef1α‐DIO‐hM4Di‐mcherry or AAV‐Ef1α‐DIO‐mCherry vector in BF of PV‐IRESCre mice. G, Imnunoflurescent of GABAParv neurons (green) and hM4Di virus expression (red), and the merged picture. H, I C‐Fos expression of BF in GABAParv‐hM4Di mice after saline or CNO i.p. injection 2 h before perfusion. Scale bar: 100 μm. J, The percent of C‐Fos positive cells in BF GABAParv nucleus. Data are presented as mean ± SEM. The percent of C‐Fos expression in GABAParv neurons with CNO pretreatment was significantly lower than that in the saline pretreatment group (*p* < 0.005, unpaired *t*‐test)

**FIGURE 6:**
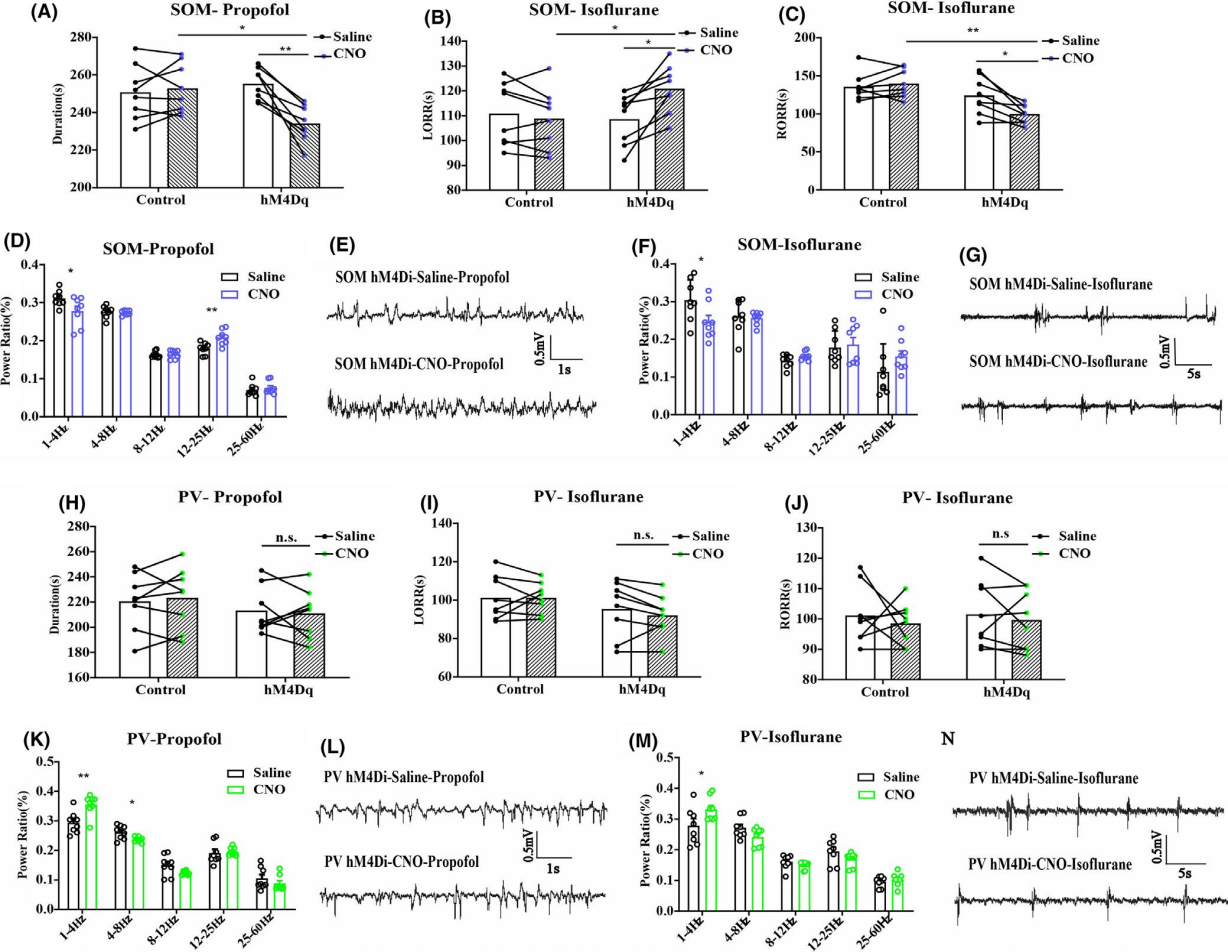
A, The duration time of propofol anesthesia in BF GABASOM neuron with hM4Di group, *n* = 8, *p < 0.05, **p < 0.005, paired *t*‐test. B, C, The LORR (B) and RORR (C) time of isoflurane anesthesia in BF GABASOM neuron with hM4Di group, *n* = 8, *p < 0.05, **p < 0.01, paired *t*‐test. D–G. The power spectrum analysis of EEG recording of mice with inactivated BF GABASOM neurons in propofol (D, E) and isoflurane (F, G) anesthesia, *n* = 8, *p < 0.05, **p < 0.005, two‐way ANOVA. H. The duration time of propofol in BF GABAParv neuron with hM4Di group, *n* = 8, n.s., no significant, paired *t*‐test. I, J, The LORR (I) and RORR (J) time of isoflurane anesthesia in BF GABAParv neurons with hM4Di group, *n* = 8, n.s., no significant, paired *t*‐test. K–N, The power spectrum analysis of EEG recording of mice with inactivated BF GABASOM neurons in propofol (K, L) and isoflurane (M, N) anesthesia, *n* = 8, *p < 0.05, **p < 0.005, two‐way ANOVA

To reversibly inactivate the BF GABA^SOM^ or GABA^Parv^ neurons, we injected AAV‐Ef1α‐DIO‐hM4Di‐mcherry and AAV‐Ef1α‐DIO‐mCherry virus in the transgene mice separately. The immunofluorescence images show the virus transfection of the BF GABA^SOM^ (Figure 5A,B, Figure S1C) and GABA^Parv^ neurons (Figure 5F,G, Figure S2C). The C‐Fos expression in BF GABA^SOM^ (Figure 5C–E) and GABA^Parv^ neurons (Figure 5H–J) with CNO pretreatment was significantly lower than that with saline pretreatment (Figure 5E,F, GABA^SOM^ 18.7% to 0.3%; GABA^Parv^ 20.0% to 1.2%). In the BF GABA^SOM^ neurons inhibited mice, the duration time of propofol was shortened (Figure 6A, control group 250.75 ± 5.70 s to 252.88 ± 8.31 s, *p* = 0.76; hM4Di group 255.25 ± 4.22 s to 234.13 ± 3.97 s, *p* = 0.00037, *n* = 8, paired *t*‐test). Furthermore, they were also harder to anesthetize with isoflurane (Figure 6B,C, LORR, control group 110.88 ± 6.92 s to 108.97 ± 5.90 s, *p* = 0.75, *n* = 8; hM4Di group 108.63 ± 5.85 s to 120.88 ± 4.56 s, *p* = 0.024, *n* = 8, paired *t*‐test; RORR, control 135.63 ± 6.83 s to 139.75 ± 7.62 s, *p* = 0.66; hM4Di group 124.00 ± 9.80 s to 99.75 ± 5.42 s, *p* = 0.027, *n* = 8, paired *t*‐test). Moreover, the slow‐delta power bands of the EEG during anesthesia showed the similar effect by the inhibition (Figure 6D–G, Propofol, hM4Dq group, 0.32 ± 0.008 to 0.27 ± 0.02, *p* = 0.038, *n* = 8; Isoflurane, hM4Di group, 0.31 ± 0.02 to 0.25 ± 0.02, *p* = 0.029, *n* = 8, two‐way ANOVA). There were no significant behavioral differences in the GABA^Parv^ neurons inactivated mice with propofol and isoflurane anesthesia (Figure 6H–J). However, a few differences were found in the delta power bands of the cortical EEG (Figure 6K–N). These results illustrate that GABA^SOM^ neurons in the BF promote propofol and isoflurane anesthesia, similar to the sleep‐promoting function in natural sleep–wake cycle. However, the GABA^Parv^ neurons did not appear to have obvious effect in anesthesia.

## 4 DISCUSSION

Our study aimed to clarify whether propofol and isoflurane induce unconsciousness mediated by BF GABA^SOM^ and GABA^Parv^ neurons. We found that chemogenetic activation or inactivation of BF GABA^SOM^ neurons affected both behavioral and EEG under propofol or isoflurane anesthesia. BF GABA^SOM^ neurons, the sleep‐promoting neuronal population in the BF,^10^ also displayed a hypnosis‐promoting effect in general anesthesia. The mechanism of general anesthesia induced consciousness transition resembled that of sleep–wake modulating circuits in brain. However, the negative results of the BF GABA^Parv^ neurons in our experiments showed that not all regions or neurons functioning in natural sleep are involved in general anesthesia.^1^, ^4^ This finding is in accordance with other studies. Therefore, it is clear that there are different regulatory elements between narcotism induced by general anesthesia and natural sleep.^24^

When destroyed BF GABA^SOM^ neurons, we found fewer EEG delta power bands but an increasing narcotism effect (longer LORR and shorter RORR time) under isoflurane anesthesia procedure. A previous experiment demonstrated that if the amount of wakefulness increased during first 4 h of the dark period (7_PM_–11_PM_), this was at expense of both NREM and REM sleep, but no significant cortical EEG differences during other periods of a day when chronically ablate GABA^SOM^ neurons in the BF.^19^ Although we completed all the experiments during the light period (7_AM_–6_PM_) to avoid this influencing factor, the sleep fragmentation might still exist.^25^ Furthermore, several experiments have shown that general anesthetics can help recover the loss of sleep in clinic and laboratory to some extent.^26^, ^27^, ^28^ Accordingly, we suspected that the increased sensitivity to general anesthetics in BF GABA^SOM^ neurons lesioned mice appears to be due to the accumulation of sleep debt, just like the increased efficacy of general anesthetics after 24 days of lesions of the ventrolateral preoptic nucleus, which is a classic sleep‐promoting brain area.^29^ Furthermore, the cortical EEG, with decreasing delta power and increasing gamma power, indicates that the destruction of BF GABA^SOM^ neurons decreased the depth of anesthesia and further suggests that general anesthetics may induce or maintain anesthesia by acting on these neurons or the circuits these neurons involved in. The results of genetic inhibition of BF^SOM^ neurons, which ruled out the accumulation of sleep debt caused by chronic ablation, more accurately prove that BF^SOM^ neurons play a role anesthetic. This also suggests that the lesion of a sleep‐promoting nucleus may affect the accuracy of our results due to sleep rebound and other reasons. Then, we did not destroy the neurons completely, and the remaining neurons might have compensatory effect in our experiment as well. Another, in the DREADDs experiment, we injected AAV‐DIO‐hM3Dq/hM4Di‐mCherry into BF area which resulted in a specific combination between hM3Dq/ hM4Di‐mCherry and somatostatin neurons. The mCherry positive neurons indicate somatostatin neurons. The green fluorescent neurons stained by somatostatin neurons antibody can merge well with mCherry fluorescent neurons, which mutually proved the accuracy of the experimental model and somatostatin immunostaining. The ratio of the colocalization neurons to BF^SOM^ neurons is 86.33 ± 1.68% in M3 group and 85.17 ± 1.70% in M4 group, and the ratio of the colocalization neurons to mCherry labeled neurons is 89.67 ± 1.52% in M3 group and 88.83 ± 1.20% in M4 group, which indicated the equal level of the virus expression in two groups.

The different functions of BF GABA^Parv^ and GABA^SOM^ neurons in the sleep–wake cycle and anesthesia may result from their separate long‐range connections. GABA^Parv^ neurons are more unidirectional, while GABA^SOM^ neurons are more reciprocal.^30^ Recent studies have shown that BF GABA^Parv^ neurons promote wakefulness^10^ and preferentially enhance cortical gamma band oscillations.^31^ The BF GABA^Parv^ neurons received mostly anatomical inputs from the nucleus accumbens (NAc), which was considered emotional valence.^32^ Moreover, behavioral adaptations require nucleus^33^ and also receive promotions from other regions like lateral hypothalamic area (LHA), ventral tegmental area (VTA), and periaqueductal gray (PAG). The major anatomical outputs of BF GABA^Parv^ neurons are the local ventral pallidum (VP) and hypothalamus.^30^, ^34^, ^35^ However, few studies demonstrated the functional connections of BF GABA^Parv^ neurons and other sleep–wake regulating regions. Synthetically, BF GABA^Parv^ neuron were reported to have a slight activating effect in the sleep–wake cycle,^10^ which is in accordance with our results that the manipulation of GABA^Parv^ neurons did not alter the process of general anesthesia, suggesting that these neurons may be not critical in arousal and/or wakefulness maintaining. Nevertheless, a more significant influence might exist if we manipulated the circuits that BF^Parv^ neurons involved in, as they have some connections to other important areas, like thalamic reticular nucleus (TRN), cingulate cortex, neocortex, etc.^36^, ^37^, ^38^ Furthermore, the technical limitations might cause some influences, such as the incompletely ablation of the target neurons might induce the compensatory effect and caused the results seen in the lesioned groups. In addition, we recorded EEG without EMG of mice synchronically and did not analyze the NREM and REM sleep separately under anesthesia, which might neglect some changes when manipulate these neurons.

Numerous evidences indicated that GABA^SOM^ neurons contain diverse regional populations and functions in the BF. GABA^SOM^ neurons in VP of BF regulate local gamma oscillations to drive prefrontal cortical activity.^39^, ^40^ In sleep–wake cycle, BF GABA^SOM^ neurons exert a sleep promoting effect,^10^ and the synthetic SOM analog, octreotide, can either suppress NREM sleep or increase REM sleep.^41^, ^42^ Furthermore, they can also promote anxiety emotion and the intake of high‐calorie food.^43^ The projections from the amygdala to different BF (VP/SI) subregions share certain organizational features with prefrontal cortical, and there are hippocampal projections to the medial septum and SI areas in the BF as well.^34^, ^44^, ^45^ In our study, we did not separate these subregions in detail. Rather, according to the slice immunofluorescence, the region we injected the virus in were mostly the horizontal limb of the diagonal band (HDB), magnocellular preoptic nucleus (MCPO), and VP. We found that the SOM neurons in these subregions were involved in anesthesia induced by isoflurane and propofol, and activation of these neurons increased the sensitivity to anesthetics, prolonged anesthesia, and synchronized cortical EEG. Future study should divide these neurons into different subgroups according to different subregions, such as the wake‐active Kv2.2‐expressing GABAergic neurons.^46^, ^47^ Additionally, some experiments indicated that the GABAergeic system could be affected by anesthetics, and this effect is related to age.^48^, ^49^ Therefore, in our study, we only included young mice (8–12 weeks) to avoid any possible effects caused by aging.

In addition, the BF not only connects with other brain regions but also contains local neuronal connectivity. The sleep‐promoting GABA^SOM^ neurons inhibit other three types of neurons in the sleep–wake cycle.^10^ The increased effect of anesthetics may partly be caused by inhibition of other active neurons in the BF and relevant regions when BF GABA^SOM^ neurons are activated. Although BF^Parv^ neurons have some connections to other important areas, they appeared to have little effect on the narcotism induced by anesthetics. Thus, our results suggest that the unconsciousness induced by general anesthetics is principally achieved by acting on a specific neural network that is involved in consciousness maintenance.

## CONFLICT OF INTEREST

The authors declare that there are no conflicts of interest in the authorship or publication of the contribution.

## Supporting information

Fig S1

Fig S2

## Data Availability

The data that support the findings of this study are available from the corresponding author upon reasonable request.
