# Supplementary figures and images for: Effect of basal forebrain somatostatin and parvalbumin neurons in propofol and isoflurane anesthesia
Source: CNS Neurosci Ther. 2021 Mar 25;27(7):792–804. doi: 10.1111/cns.13635 (PMC8193699; doi:10.1111/cns.13635)

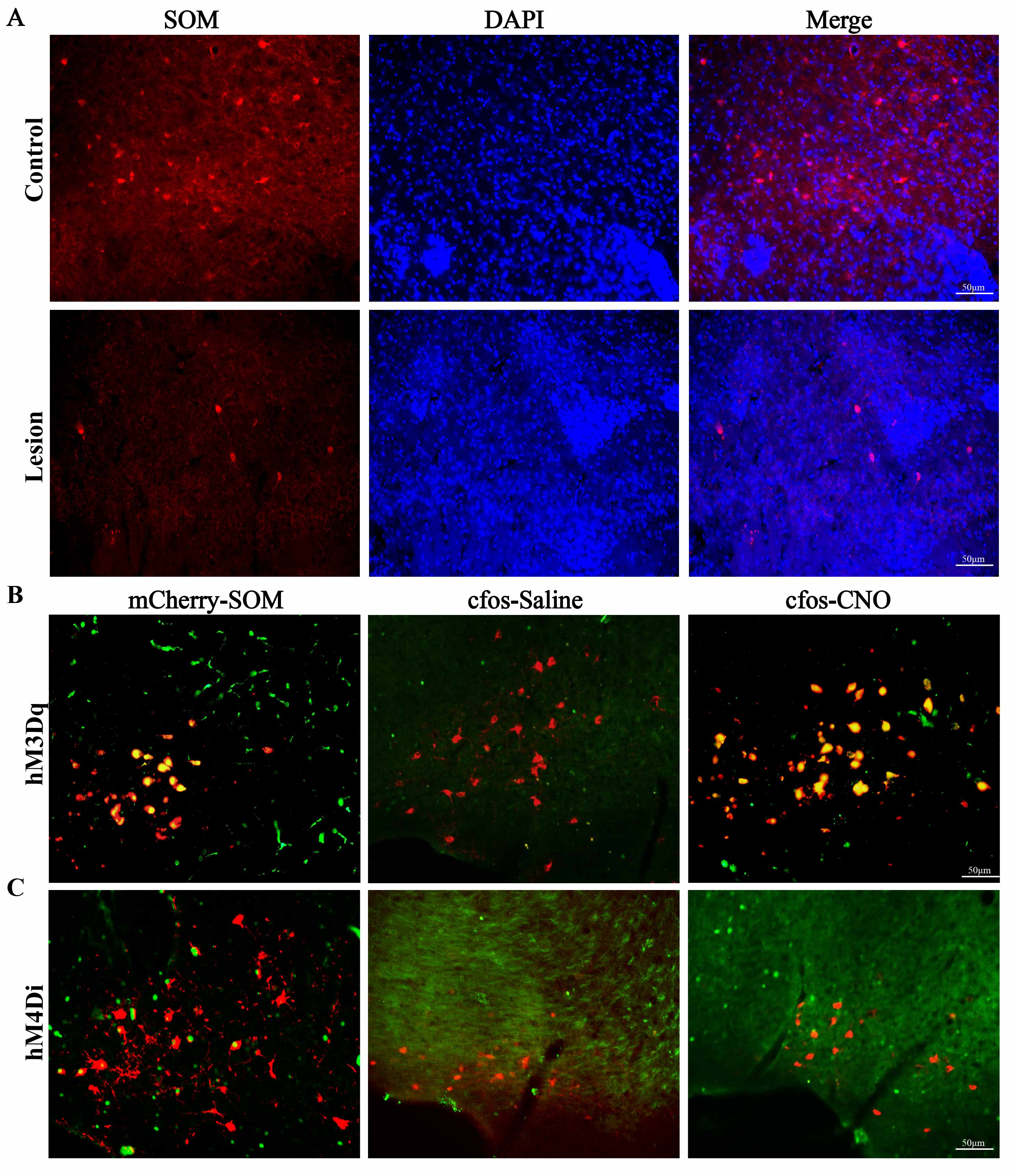

Supplement: Supplementary file 1 — Fig S1 [file CNS-27-792-s001.jpg]

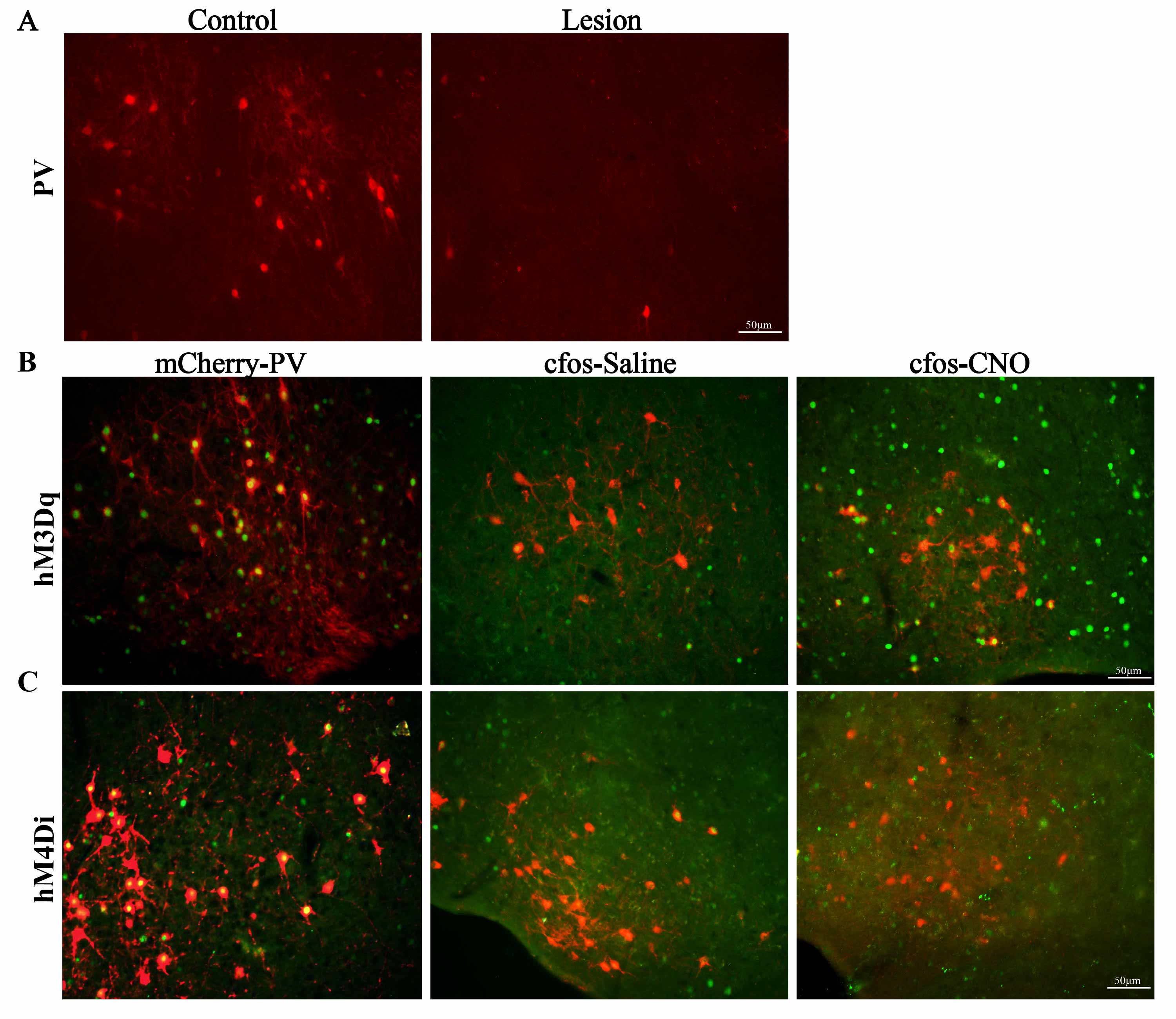

Supplement: Supplementary file 2 — Fig S2 [file CNS-27-792-s002.jpg]
